# Dietary supplementation with *Tolypocladium sinense* mycelium prevents dyslipidemia inflammation in high fat diet mice by modulation of gut microbiota in mice

**DOI:** 10.3389/fimmu.2022.977528

**Published:** 2022-11-07

**Authors:** Xiaolong Wang, Lin Li, Mingjian Bai, Jiaxin Zhao, Xiaojie Sun, Yu Gao, Haitao Yu, Xia Chen, Chunjing Zhang

**Affiliations:** ^1^ Department of Medical Technology, Qiqihar Medical University, Qiqihar, Heilongjiang, China; ^2^ National & Local United Engineering Laboratory for Chinese Herbal Medicine Breeding and Cultivation, School of Life Sciences, Jilin University, Changchun, China

**Keywords:** *Tolypocladium sinense*, gut microbiome, dyslipidemia, inflammation, obesity

## Abstract

Obesity is a risk factor for many serious health problems, associated with inflammation, hyperlipidemia, and gut dysbiosis. Prevention of obesity is especially important for human health. *Tolypocladium sinense* is one of the fungi isolated from Chinese caterpillar fungus, which is a traditional Chinese medicine with putative gut microbiota modulation effects. Here, we established a high-fat diet (HFD)-induced hyperlipidemia mice model, which was supplemented with lyophilized *T. sinense* mycelium (TSP) daily to evaluate its anti-obesity effects. The results indicated that TSP supplementation can effectively alleviate the inflammatory response and oxidative stress levels caused by obesity. TSP significantly prevented obesity and suppressed dyslipidemia by regulating the expression of lipid metabolism genes in the liver. TSP is also effective in preventing the HFD-induced decline in short-chain fatty acid (SCFA) content. Gut microbiota profiling showed that TSP supplementation reversed HFD diet-induced bacterial abundance and also altered the metabolic pathways of functional microorganisms, as revealed by KEGG analysis. It is noteworthy that, correlation analysis reveals the up-regulated gut microbiota (*Lactobacillus* and *Prevotella_9*) are closely correlated with lipid metabolism parameters, gene expression of liver lipid metabolism and inflammatory. Additionally, the role of TSP in the regulation of lipid metabolism was reconfirmed by fecal microbiota transplantation. To sum up, our results provide the evidence that TSP may be used as prebiotic agents to prevent obesity by altering the gut microbiota, alleviating the inflammatory response and regulating gene expression of liver lipid metabolism.

## Introduction

Non-communicable Disease Risk Factor Collaboration reported that the global rate of the prevalence of the age-standardized obesity increased approximately 2-3 times in 2014 compared with that in 1975. Approximately 1.9 billion people in the world are overweight, and among them, 600 million are obese. Obesity has become a serious hazard to human health, it can induce diabetes, non-alcoholic fatty liver disease, hypertension and certain (1, 2). Multiple factors contribute to the development of obesity, including energy consumption, high fat intake and the microbiome (3). Many reports reveal that gut microbiota acts an important modulator in the diet and metabolic syndrome is caused by obesity (4, 5). Diet is a significant factor altering the diversification and metabolism of the gut microbiota, consequently inducing or preventing obesity (6, 7).

The over consumption of food in the host and consequent increase in energy intake is the main cause of obesity; the intestinal flora is involved in the regulation of nutrient absorption and energy balance. The results of some basic studies showed that the intestinal permeability of obese mice is significantly enhanced, the diversity of the intestinal flora is reduced, Bacteroidetes decrease by approximately 50%, and the number of Firmicutes increased in proportion, as compared with lean mice (8, 9). The results of a clinical research show that Bacteroides ferment dietary fibers to produce short chain fatty acids, while Firmicutes obtain energy from food and store it in the form of adipose tissue (10). Studies in animal models suggest that certain gut microbes can prevent diet-induced obesity. Indeed, several probiotics have been used in clinical trials to reduce lipid levels in obesity-regulated subjects, achieving good results (11–13).

Chinese caterpillar fungus is a traditional Chinese medicinal mushroom, which contain a wide range of immuno-modulatory and bioactive compound with many medical effects, such as anti-aging, anti-bacteria, anti-cancer, expanding blood vessels, improving arteriosclerosis, hepatoprotective and hypolipidemic (14). *Tolypocladium sinense* is one of the fungi isolated from Chinese caterpillar fungus. The research and application of *T. sinense* mainly focus on the culture conditions and the preliminary pharmacological analysis of its chemical components (15, 16). Fang (17) carried out pharmacological experiments on the mycelium culture of *T. sinense* in mice. The results showed that it possesses sedative effects, anti-inflammatory activity, hypoxia tolerance, organ expansion and androgen like promotion. In the acute toxicity test, the dose of 80 g (maximum allowable volume) per mouse was administered once by gavage, and no adverse effects were found. Gao (18) reported that the mycelium extract and polysaccharide extract of *T. sinense* possess scavenging effects on DPPH free radicals. The test results showed that *T. sinense* has potential application and development prospects as antioxidant and anti-tumorigenic.

At present, the research on the pharmacological value of *T. sinense* is not complete, since its role in preventing obesity and its ability to change the gut microbiota composition is still unclear. Therefore, the purpose of our study was to determine the effects of *T. sinense* mycelium (TSP) in the prevention of hyperlipidemia and to understand its potential lipid-lowering mechanism. This study could provide a theoretical basis for the development of prebiotic agents to prevent obesity from a Chinese traditional edible fungus.

## Materials and methods

### Materials and reagents

Serum biochemical detection index kit such as total cholesterol (TC), triglyceride (TG) and ELISA detection kit were purchased from Nanjing Jiancheng Institute of Bioengineering (Jiangsu, China). Blood glucose assay kit was obtained from Jiangsu Yuyue Medical Equipment & Supply Co., Ltd. (Jiangsu, China). All other chemical reagents were analytical grade.

### Preparation and identification of *Tolypocladium sinense* fungus powder

Natural fresh Chinese caterpillar fungus was collected in the plateau area at an altitude of 4000 ~ 4500 m in the Naqu, Tibet Autonomous Region. The fungus was thoroughly rinsed with tap water. Then it was submerged in 10% bleach water for 20 min and rinsed with sterile distilled water. The fruiting bodies of Chinese caterpillar fungus were cut into small pieces of 2 ~ 5 mm and cultured on a separation medium (10 g/L peptone, 100 g/L glucose, 3 g/L yeast extract, 0.5 g/L MgSO_4_, 1 g/L KH_2_PO_4_, 100 U/L penicillin, and 20 g/L agar) at 26 °C. After the grow of the mycelium, the tip was collected and inoculated on fresh medium. The separation and passages were repeated several times until the colonies with consistent morphology were obtained (18, 19). The strain identification was performed as follows: the mycelial DNA was extracted, the whole genome was used as the template, and the universal primers ITS1 and ITS4 as primers **(** **Table S1** **)** were used for PCR amplification. The amplified products were sequenced and analyzed by Shanghai Sangon Biotech Co., Ltd. The strain screened by morphological observation and sequencing identification was *T. sinense*. After the identification of the strain, the metabolites of the bacterial mycelium were analyzed by Beijing BioMarker Technology Co., Ltd. (**Supplemental Methods** and **Figure S1**). In total, we detected 1652 metabolites from *T. sinense* fungus mycelium, and most of them belong to Organic acids, Nucleic acids, Glycerophospholipids, Fatty Acyls, Organoheterocyclic compounds, Carbohydrates, Polyketides, Organic oxygen compounds and Sterol Lipids. Pick up the cultured colonies, connect with 5% seed culture medium for culture, shake at 26 °C for 4-5 days (150 r·min^-1^), centrifuge the obtained fermentation culture medium at 4000 r · min^-1^ for 10 min, take the precipitation and freeze-dry to obtain the dried mycelium powder.

### Animals and diet

Six-week-old male C57BL/6 mice weighing 20.0 ± 1.0g were provided by the experimental animal center of Qiqihar Medical University (SYXK (HEI) 2016-001). The mice were then randomly divided into the following groups (n = 8 per group): NC group, in which the mice were fed with a standard diet (total calories: 4.3 kcal/g, 10 kcal% fat); HFD group, in which the mice were fed with a high-fat diet (total calories: 6.1 kcal/g, 60 kcal% fat); TSP group, in which the mice were fed with a high-fat diet supplemented with *T. sinense* mycelium (400 mg·kg^-1^·day^−1^). Animal weight and food intake were recorded weekly during the study. Fresh feces were collected in a separate sterile EP tube after 10 weeks and stored at − 80°C for subsequent microbiota analysis. The mice were sacrificed after fasting overnight. Liver tissue, fat pad and blood samples were collected. Serum was obtained by centrifugation (1200 g, 15 min) and stored at -80°C for further study. Serological analysis and histology were described in the supplementary data.

### TSP treatment for antibiotic-treated mice

The male C57BL/6J mice aged 6 weeks (20.0 ± 2.0g) were fed with the NC-diet and treated with antibiotics (0.5 g/L vancomycin, 1.0 g/L ampicillin, 1 g/L metronidazole, 1 g/L zincomycin sulfate) to establish pseudo germ-free mice, mixed antibiotics diluted daily with distilled water for drinking (20). After 14 days of antibiotics treatment, the microbiota-depleted mice were randomly allocated into three groups, MTNC, MTHFD and MTTSP (n=12/group) which were transplanted with the microbiota from mice fed with NC, HFD, and TSP (treated for 10 weeks) respectively. In detail, every 200 mg of pollution-free feces was added into 5 mL PBS/DTT sterile solution to a 5 mL sterile EP tube, which was shaken and rotated for 2 min in anaerobic state (20). The impurities were removed by 100 μm sterile filter for three times. After 7 days, 4 mice were randomly selected to collect fresh feces to detect colonization by high-throughput sequencing (16SrDNA v3-v4). The results are shown in the supplementary data (**Figure S2**). Then half of the mice in each microbiota transplanted group fed with the NC diet and the other half fed with HFD for 15 days. Then, blood, tissues and feces were collected for analyses.

### Quantitative real-time PCR

Total RNA from hepatic tissue was isolated using Biozol reagent (Invitrogen Carlsbad, CA, USA) by a method previously described (21), and the concentration was determined by NanoDrop spectrophotometer (BioTeke, Beijing, China). cDNA was synthesized using a reverse transcriptase Kit (manufacturer) according to the manufacturer’s instructions. SYBR Green real-time (TransGen Biotech, Beijing, China) was used for quantitative PCR in real time. The primer sequences used in this study are listed in **Table S1** of the supplementary data. The quantification of the target genes was performed using the 2^-△△^Ct method (22) using β-actin as the reference gene and the NC group as control.

### Short chain fatty acids analysis

The concentration of short chain fatty acids (SCFAs) was measured by gas chromatography (GC) as previously described with some modification (23, 24). Feces were collected from each rat, 2 g into were placed into a sterile centrifuge tube, and 1 ml methanol solution was added. The tube was left standing for 10 min, then it was shaken and well mixed to form a fecal suspension. Then, a concentrated sulfuric acid was used to adjust its pH to 2 ~ 3, the tube was left standing for 5 min, and then it was shaken and mixed several times. Next, the tube was centrifuged at 5000 r·min^-1^ for 20 min, the supernatant was collected and centrifuged at 5000 r·min^-1^ for 5 min, and the supernatant was collected and placed into the gas chromatograph for the analysis. An Agilent kit was used to determine the content of short chain fatty acids. The gas chromatograph 7890a used in this analysis was equipped with a flame ionization detector. The da-ffap column (30 m × 0.320 mm × 0.25 μ m) was used to separate short chain fatty acids. The parameters of the gas chromatograph were the following: temperature of injection port, 250°C; nitrogen as carrier gas, with purity ≥ 99.99%; carrier gas flow rate, 30 mL·min^-1^ injection mode, split injection; split ratio, 50:1; injection volume, 1 μL; detector temperature 250°C; temperature rise procedure, 80°C, 10°C·min^-1^, 180°C.

### Gut microbiota analysis

Genomic DNA was extracted using the MOBIO PowerSoil^®^ DNA Isolation Kit (MOBIO, UnitedStates), and the concentration was determined by NanoDrop spectrophotometer (BioTeke, Beijing, China). A total of 10 ng DNA template was used for PCR amplification according to the sequence of 16SrDNA v3-v4 region with specific primers 338F/806R. Truseq^©^ DNA PCR-Free Sample Preparation Kit was used to construct the library. The constructed library was quantified by qubit and qPCR. After the library was qualified, the sequencing was carried out on Illumina Novaseq 6000 platform according to the manufacturer’s instruction. The sequencing was completed by Beijing Bio Marker Technology Co., Ltd. Usearch software (25) was used to cluster the reads at 97.0% similarity level and OUT was obtained. SILVA was used as the reference database, using naive Bayesian classifier combined with comparison method to annotate the feature sequence. The species classification information corresponding to each feature was obtained, then the community composition of each sample at each level (phylum, class, order, family, genus, species) was counted, and the species abundance at different classification levels was generated by the QIIME software. Then, the community structure map of each taxonomic level of the sample was drawn by R software (Version 3.4.1). Non-Metric Multi-Dimensional Scaling(NMDS);adopts Bray Curtis algorithm; Lefse (26) (line discriminant analysis (LDA) effect size) was used to find biomarkers with statistical differences between different groups.

### Serologic and hepatic index analysis

The concentrations of total triglyceride (TG), cholesterol (CHO), low density lipoprotein (LDL-C), non-esterified fatty acid (NEFA), malondialdehyde (MDA), superoxide dismutase (SOD), glutathione peroxidase (GSH-Px), tumor necrosis factor- α (TNF-α), interleukin-6 (IL-6) and interleukin-1β (IL-1β) in serum and hepatic carried out in strict accordance with the instructions of the kit (Nanjing Jiancheng Bioengineering Institute, Nanjing, China).

### Histological analysis

The hepatic of mice in each group were dissected and extracted and fixed with 4% paraformaldehyde. After the fixation was in good condition, they were trimmed, dehydrated, embedded, sliced, stained, sealed, sliced, stained with Hematoxylin eosin (HE), and the structure of liver tissue was observed and analyzed under optical microscope, as it was previously described (27).

### Statistical analysis

Statistical analysis was performed using SPSS 20.0 software. Statistical differences among different groups were analyzed by one-way analysis of variance (ANOVA) followed by Tukey– Kramer *post hoc* test. Other statistical tests for significance were performed using R software (Version 3.4.1) for windows. Results were expressed as mean± SD. A value of *p* < 0.05 was considered statistically significant.

## Results

### TSP supplementation alleviated HFD-induced weight gain and fat accumulation in mice

Our preliminary animal experiment was performed using TSP at the doses of 100, 200, and 400 mg·kg^-1^ ·day^−1^. The medium dose and high dose exerted a significant prevention of the abnormal lipid metabolism compared to HFD group (*p<* 0.05; **Table S2**), while the low dose had no significant effect compared to the HFD group. The effect of the high dose was more remarkable than that of the medium dose. Therefore, 400 mg·kg^-1^·day^−1^ of TSP supplementation was the dose used in this study.

**Figure 1:**
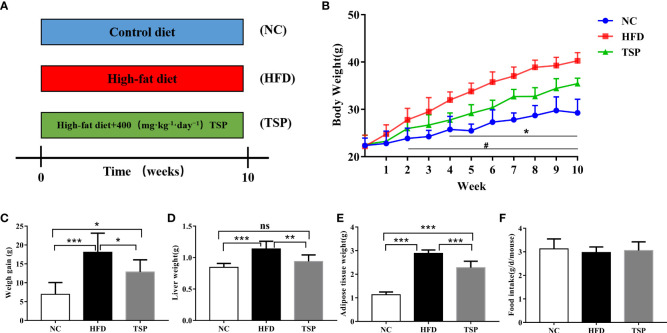
Effects of TSP consumption on the **(A)**the experimental protocol used in this study, n=8, **(B)** body weight ^#^ *p* < 0.05, NC compare with HFD, *p < 0.05, TSP compare with HFD, **(C)** body weight gain, **(D)** liver weight **(E)** adipose tissue weight, **(F)** food intake. Data are expressed as means ± SD (*n* = 8). ^#/^*p < 0.05, **p < 0.01, and ***p < 0.001, ns, no significant *p* > 0.05.

During the 10-week experimental period (**Figure 1A**), the average body weight of the NC mice group at week 10 was 29.27 ± 2.89 g, and that of the HFD mice group was 40.28 ± 1.73 g (*p* < 0.05 versus the NC group, **Figure 1B**). The increase in the body weight of the TSP group was significantly suppressed compared with the HFD group (*p*< 0.05). Consistently, the body weight gain, liver weight and adipose tissue weight was lower in the TSP group than that in HFD group (**Figures 1C–E**). No differences were observed in the daily food intake among the three groups (**Figure 1F)**.

**Figure 2:**
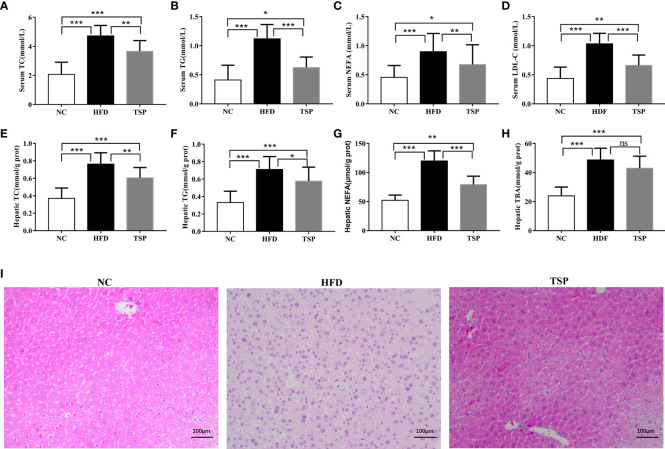
Effects of TSP supplementation on the serum and hepatic **(A, E)** total cholesterol (TC), **(B, F)** triglyceride (TG), **(C, G)** non-esterified fatty acid levels (NEFA), **(D)** low-density lipoprotein cholesterol (LDL-C) in serum, **(H)** Hepatic total bile acid (TBA),**(I)** H&E staining of mouse livers. Values are expressed as mean ± SD in each group (n = 8), *p < 0.05, **p < 0.01, and ***p < 0.001, ns, no significant *p* > 0.05.

Additionally, HFD induced hepatic fat accumulation and dyslipidemia could also be prevented by TSP supplemented, as indicated by the serum levels of total cholesterol (TC), triglycerides (TG), non-esterified fatty acids (NEFA) and low-density lipoprotein-cholesterol (LDL-C) in the HFD-fed mice sharply increased when compared with the NC group (*p*< 0.05, **Figures 2A–D**). In addition, the HFD-fed mice were characterized by higher levels of TC, TG, NEFA and total bile acid (TBA) in the liver (*p*< 0.05, **Figures 2E–H**). TSP supplementation significantly prevented these adverse changes expect TBA in the HFD-fed mice. H&E staining showed less ballooning degeneration in the TSP group than in the HFD group **Figure 2I**).

**Figure 3:**
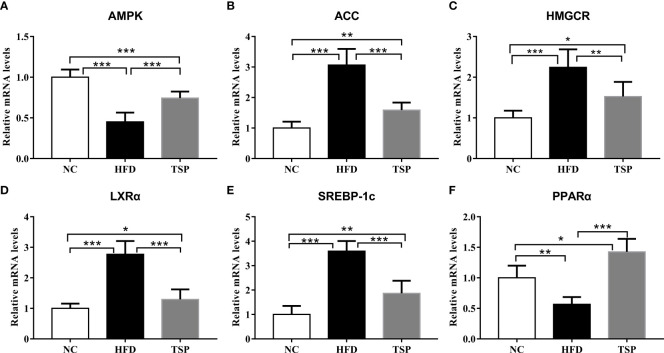
Effect of TSP on mRNA expression levels of hepatic metabolic regulators. **(A)** AMPK, **(B)** ACC, **(C)** HMGCR, **(D)** LXRα, **(E)** SREBP-1c, **(F)** PPARα. Data are expressed as means ± SD (n = 8), *p < 0.05, **p < 0.01, and ***p < 0.001.

To explore the mechanism of TSP in lipid metabolism, we examined the expression of genes related to lipid metabolism in the liver by qRT-PCR (**Figure 3**). Compared with the NC group, the expression of ACC, HMGCR, LXRα and SREBP-1c was significantly higher and the expression of AMPK and PPARα was significantly lower expression in the HFD group (*p*<0.05). Compared with the HFD group, TSP supplementation significantly decreased ACC, HMGCR, LXRα and SREBP-1c expression in the liver and enhanced AMPK and PPARα expression (*p* < 0.05). TSP supplementation did not affect expression of CD36, CYP7A1, FAS, Ldlγ, LXRβ or PPARγ **(** **Figure S3** **)**.

### Intake of TSP notably alleviate systematic inflammation and improve antioxidant ability in high fat diet-fed mice

**Figure 4:**
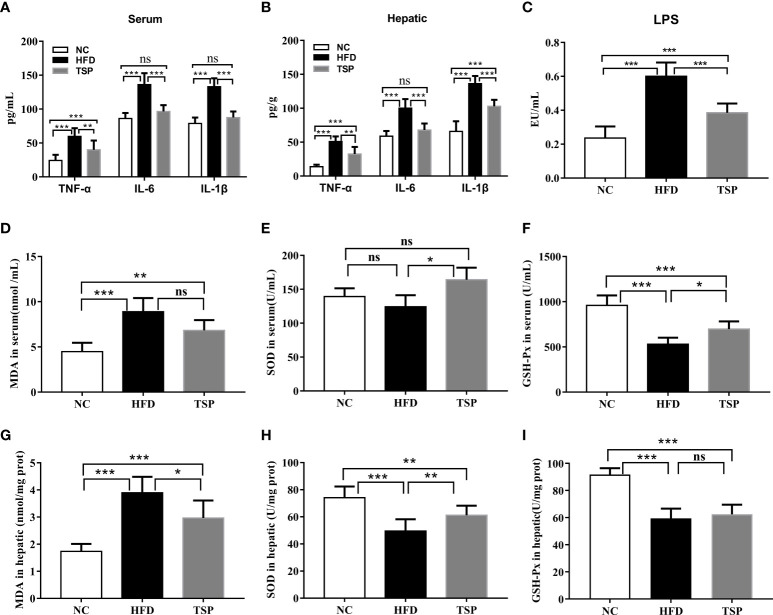
Effect of TSP on inflammation in serum **(A)** and in hepatic, **(B)** Effect of TSP on LPS, **(C)** MDA level and antioxidative enzymes (SOD, GSH-Px) in serum, **(D–F)** and in hepatic**(G-I)**. Data are expressed as means ± SD (n = 8), *p < 0.05, **p < 0.01, and ***p < 0.001, ns, no significant *p* > 0.05.

The levels of TNF-α, IL-6 and IL-1β in both serum and liver were higher in the HFD group compared to those in the NC group. The level of serum LPS showed the similar trends (**Figures 4A–C**). TSP supplementation was able to significantly reduce serum and hepatic TNF-α, IL-6, IL-1β and LPS (*p*< 0.05). Thus, TSP supplementation significantly alleviated systemic inflammation. In addition, the activity of the antioxidative enzymes (SOD, GSH-Px) and MDA level were measured in the serum and hepatic to evaluate the influence of TSP on the antioxidant ability (**Figures 4D–I**). Compared with the NC group, mice in the HFD group showed higher MDA level in serum, while the GSH-Px activity were lower in HFD group (*p* < 0.05). TSP supplementation was able to significantly reduce serum MDA level and improve SOD and GSH-Px activity (*p*< 0.05). The activity of SOD and MDA level in the liver showed the same tendency. The activity of GSH-Px has no significant differences between HFD and TSP in liver (*p* > 0.05).

### TSP supplementation increased short chain fatty acids contents in the feces

Compared with the NC group, the content of acetate, propionate, butyrate, valerate and total short chain fatty acids was decreased by 34.54%, 49.58%, 33.02%, 11.11% and 35.16%, respectively, in the HFD group (*p* < 0.05, **Table 1**). However, compared with the HFD group, an increased short chain fatty acids level was observed by TSP supplementation in the TSP group (*p* < 0.05).

**Table 1:** Effect of TSP supplementation on the concentrations of acetate, propionate, butyrate, valerate and total SCFAs in the feces.

SCFAs(μmol/g)	NC	HFD	TSP
Acetate	37.69 ± 4.45a	24.67 ± 4.76b	40.21 ± 5.77a
Propionate	2.38 ± 0.31a	1.20 ± 0.43b	2.56 ± 0.22a
Butyrate	3.21 ± 0.74a	2.15 ± 0.50b	4.29 ± 0.84c
Valerate	0.18 ± 0.04a	0.16 ± 0.04b	0.20 ± 0.05a
Total SCFAs	43.46 ± 4.29a	28.18 ± 4.81b	47.26 ± 6.21a

Significant differences (*p* < 0.05) are indicated using different letters (a, b, c)

### TSP modulated composition and function of gut microbiota at different taxonomic levels

**Figure 5:**
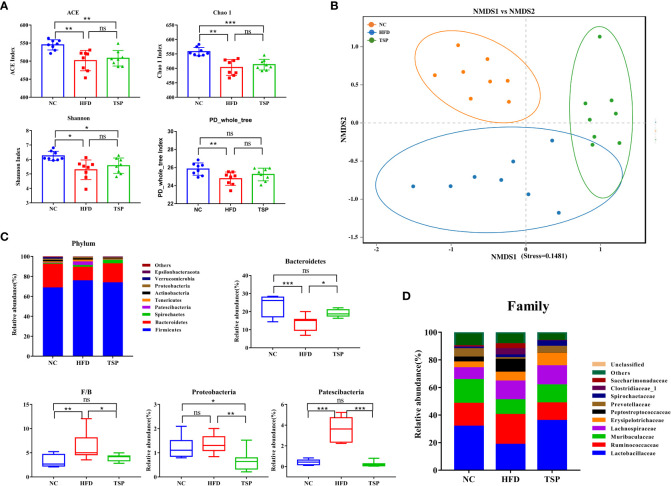
Effect of TSP supplementation on diversity and structure of the gut microbiota. **(A)** Alpha diversity analysis of ACE, Chao1, Shannon and PD-whole-tree index **(B)** Non-metric multidimensional scaling (NMDS) result based on Bray Curtis algorithm. **(C)** significantly changes (*p* < 0.05) of the composition of the gut microbiota at phylum taxa level. **(D)** Changes of the composition of the gut microbiota at family taxa level. Data are expressed as mean ± SD, *p < 0.05, **p < 0.01, and ***p < 0.001, ns, no significant *p* > 0.05.

The gut microbiota composition was analyzed by Illumina MiSeq platform.After quality filtering, the 24 samples (n = 8 for each group) resulted in a total of 1,501,889 clean reads, and at least 58,512 clean reads were generated per sample. Alpha diversity reflected the community richness and microbial evenness. Changes in alpha diversity due to the TSP treatment are shown in **Figure 5A**. The results showed that the ACE index, Chao 1 index, Shannon index and PD-whole-tree index of the HFD group were significantly lower than those in the NC group (*p* < 0.05), indicating that the HFD induced a lower microbiota community diversity. TSP treatment ineffectively increased microbial richness and diversity. The Beta diversity analysis using UPGMA clustering (**Figure S4**) and NMDS on the Bray-Curtis algorithm (**Figure 5B**) showed that the NC group clustered separately from the HFD and TSP groups. The results of PERMANOVA showed a significant difference among NC, HFD, and TSP groups (*p<* 0.001, *R* ^2^ = 0.414, Stress=0.1481).

The relative abundance at phylum, family, and genus level was compared among groups to identify specific changes in the gut microbiota due to TSP supplementation. At the phylum level (**Figure 5C**), HFD induced a much lower relative abundance of Bacteroidetes than the NC group, which increased after TSP treatment (*p* < 0.05). The ratio of Firmicutes to Bacteroidetes (F/B ratio) in the HFD group was higher than that in the NC group (*p* < 0.05). TSP supplementation fully prevented HFD-induced increase in the F/B ratio, a hallmark of obesity, which is a common indicator for gut microbiota balance. Besides, the HFD group showed a much higher relative abundance of Patescibacteria than NC group, with no difference in the relative abundance of Proteobacteria between the two groups (*p* > 0.05). While, the relative abundance of Proteobacteria and Patescibacteria significantly decreased in the TSP group compared with HFD alone (*p* < 0.01).

At the family level (**Figure 5D** and **Table S3**), compare with the NC group, the HFD group showed an increase in the abundance of Peptostreptococcaceae and Saccharimonadaceae (*p* < 0.05), while TSP supplementation decreased these two genera compared with their abundance in the HFD group. HFD induced a much lower abundance of Lactobacillaceae, Muribaculaceae and Prevotellaceae and the three genera significantly increased after TSP supplementation (*p* < 0.05). The mice in the TSP group showed a lower Ruminococcaceae and Clostridiaceae_1 compared with the mice in the HFD group (*p*< 0.05).

**Figure 6:**
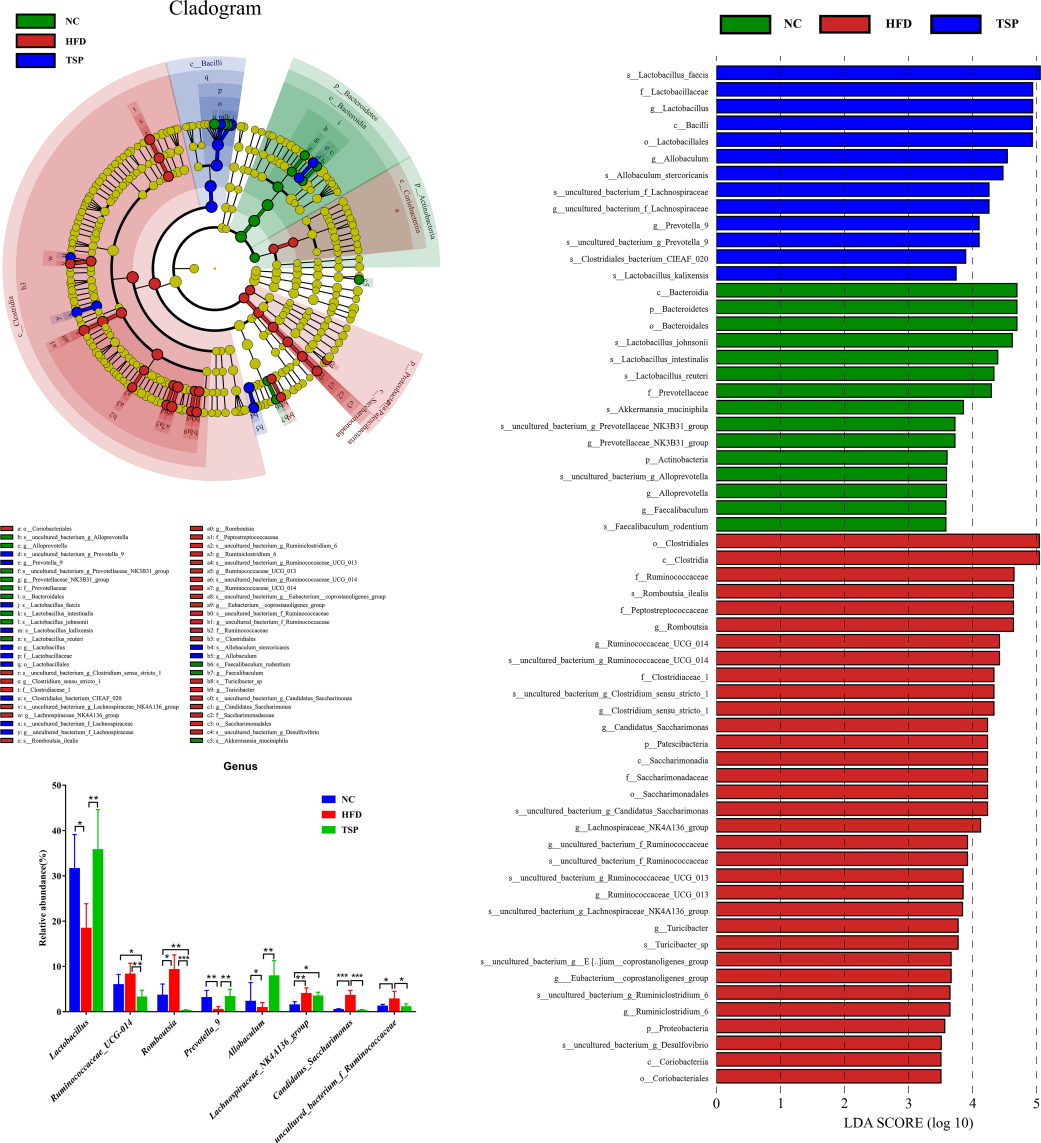
TSP supplementation induced gut microbial changes in mice. **(A)** Linear Discriminant Analysis Effect Size (LEfSe) analysis of key genera of gut microbiota in mice, **(B)** and the LDA score, **(C)** significantly changes (*p* < 0.05) among top 15 taxa of the composition of the gut microbiota at genus taxa level, *p < 0.05, **p < 0.01, and ***p < 0.001.

LEfSe (LDA sore > 3.5) was used to recognize the specific altered bacterial phenotypes at each phylogenetic level (**Figures 6A, B**) to further explore the difference in the gut microbiota among NC, HFD and TSP group. A total of 61 bacteria significantly changed among the NC group, HFD group and TSP group; they respectively showed 15, 33 and 13 dominant microorganisms. At the genus level, the main microbiota in the NC group were *Prevotellaceae_NK3B31_group*, *Alloprevotella* and *Faecalibaculum*. The result showed eleven discriminative features in the HFD group, and the main microbiota were *Romboutsia*, *Ruminococcaceae_UCG-014, Clostridium_sensu_stricto_*1, *Candidatus_Saccharimonas* and *Lachnospiraceae_NK4A136_group*. Moreover, *Lactobacillus*, *Allobaculum*, *uncultured_bacterium_f_Lachnospiraceae* and *Prevotella_9* were the main microbiota in the TSP group.

At the genus level, the microbiota with significant differences between groups were screened using Mann Whitney U test by pairwise comparison (**Figure 6C**). Collectively, the HFD group showed an increased level of *Ruminococcaceae_UCG-014*, *Romboutsia, Lachnospiraceae_NK4A136_group*, *Candidatus_Saccharimonas* and *uncultured_bacterium_f_Ruminococcaceae* compared with the NC group (*p* < 0.05). Therefore, TSP supplementation reduced the abundance of the above gut microbiota except *Lachnospiraceae_NK4A136_group* compared with HFD (*p* < 0.05), and TSP also effectively increased the relative abundance of *Lactabacillus*, *Prevotella_9* and *Allobaculum* which have a much lower abundance by HFD induced (*p* < 0.05).

### Effects of TSP supplementation on the functional change of microbial communities

**Figure 7:**
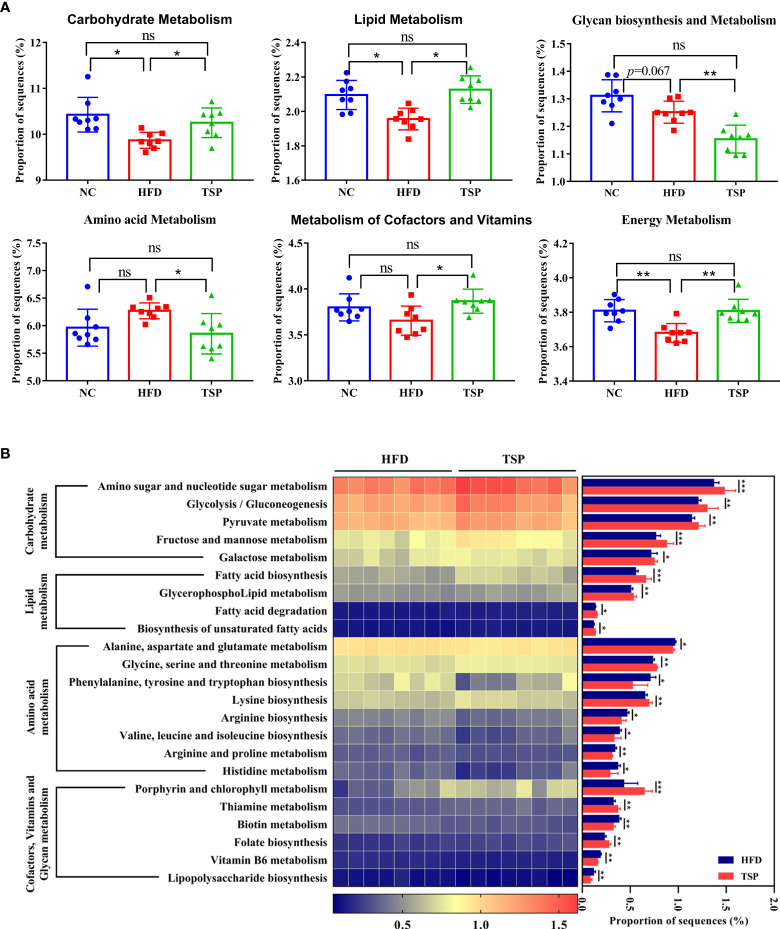
TSP supplementation induced function microbial changes in mice. **(A)** Abundances of top 6 KEGG pathways in level-2 of the functional prediction by PICRUSt, **(B)** Functional profiles with significant different between HFD and TSP treated groups. *n* = 8, *p < 0.05, **p < 0.01, and ***p < 0.001, ns, no significant *p* > 0.05.

PICRUSt analysis was carried out to explore the functional change of microbiota communities, and the comparison of top 6 metabolism category in each group is shown in **Figure 7A**. Compared with the NC group, HFD group decreased the carbohydrate metabolism, lipid metabolism and energy metabolism (*p*< 0.05), while TSP supplementation increased the proportion of carbohydrate metabolism, lipid metabolism, cofactors and vitamin metabolism and energy metabolism compared with the HFD group (*p*< 0.05).

Kyoto Encyclopedia of Genes and Genomes (KEGG) pathway showed a significant difference (*p*< 0.05) in the predictive function between the HFD group and TSP group (**Figure 7B**). Compare with HFD, carbohydrate metabolism such as amino sugar and nucleotide sugar metabolism, glycolysis/gluconeogenesis, pyruvate metabolism, fructose and mannose metabolism and galactose metabolism were significantly increased in the TSP mice (*p*<0.05). Lipid metabolism such as fatty acid biosynthesis and degradation, glycerophospholipid metabolism as well as biosynthesis of unsaturated fatty acids was increased in the TSP group (*p*< 0.05). Moreover, the amino acid metabolism pathway such as alanine, aspartate and glutamate metabolism, phenylalanine, tyrosine and tryptophan biosynthesis, arginine biosynthesis, valine, leucine and isoleucine biosynthesis and arginine, proline metabolism and histidine metabolism were decreased in the TSP group (*p* < 0.05). Only two functions of the gut microbiota in the amino acid metabolism pathway were increased in the TSP group than in the HFD group (*p*< 0.05). The functions related to cofactors and vitamin metabolism were increased in the TSP group, mainly involving porphyrin and chlorophyll metabolism, thiamine metabolism and folate biosynthesis than in the HFD group (*p*< 0.05). It is worth noting that the lipopolysaccharide biosynthesis belonging to glycan biosynthesis was decreased in TSP mice compared with HFD mice (*p*< 0.05).

### Possible relationships between reshaped gut microbiotas and biochemical changes

**Figure 8:**
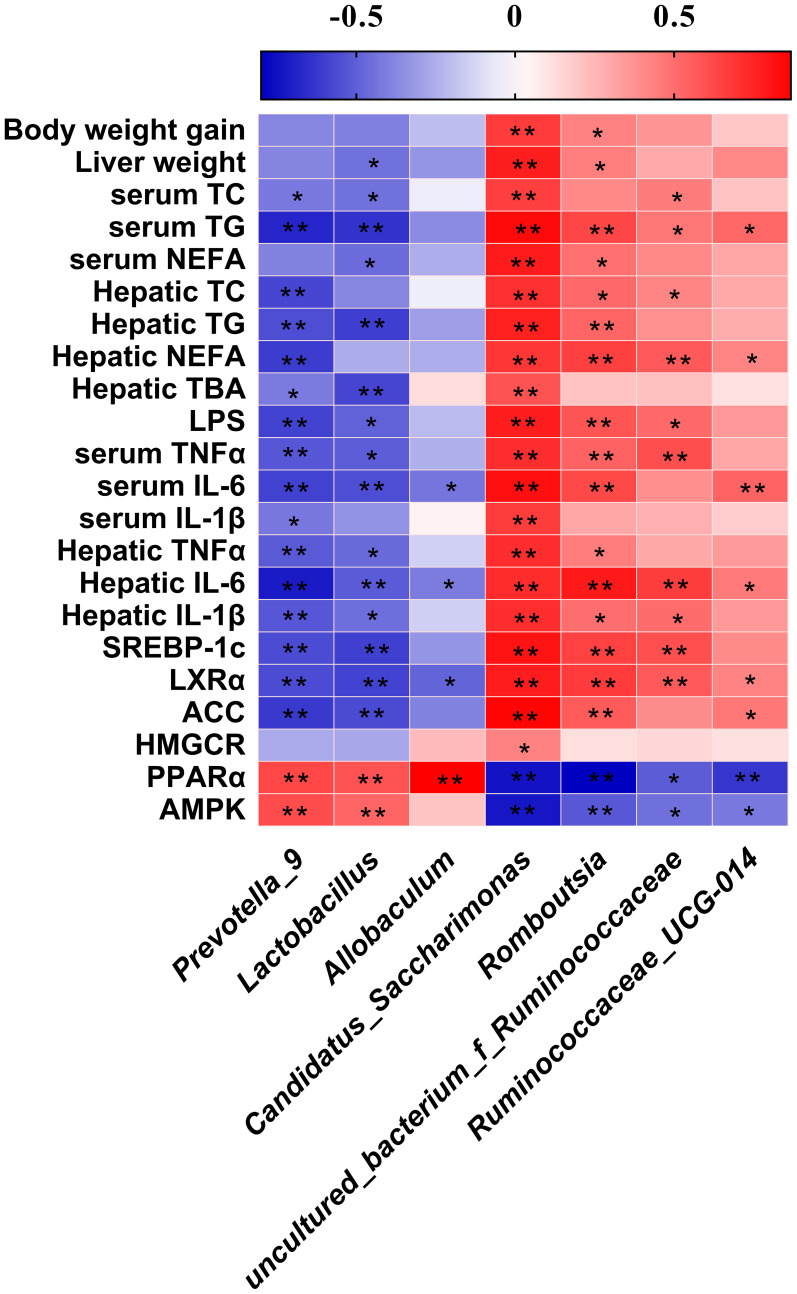
Heatmap of Spearman’s correlation between the gut microbiota and obesity-related indices. The intensity of the colors represented the degree of association (red, positive correlation; blue, negative correlation). Significant correlations are marked by *p < 0.05; **p < 0.01.

The Spearman’s correlation analysis revealed between the abundance of significantly differential bacteria at genus level identified above and parameters associated with obesity (**Figure 8**). We found that *Prevotella_9* and *Lactococcus* both showed a significant negatively correlated with parameters of lipid metabolism except body weight gain, liver weight, NEFA, IL-1β in serum and TC, NEFA, HMGCR expression in liver, and significant positive correlation with the expression of PPARα, AMPK. *Allobaculum* has the same correlation trend. *Candidatus_Saccharimonas* and *Romboutisia* both showed a significant positive correlation with parameters of lipid metabolism except TC, IL-1β in serum and TBA, HMGCR expression in liver. The genus *uncultured_bacterium_f_Ruminococcaceae* and *Ruminococcaceae_UCG-014* had the same correlation trend.

### Microbiota transplantation from TSP- supplementation mice exerts an anti-obese Effect in HFD-fed mice

**Figure 9:**
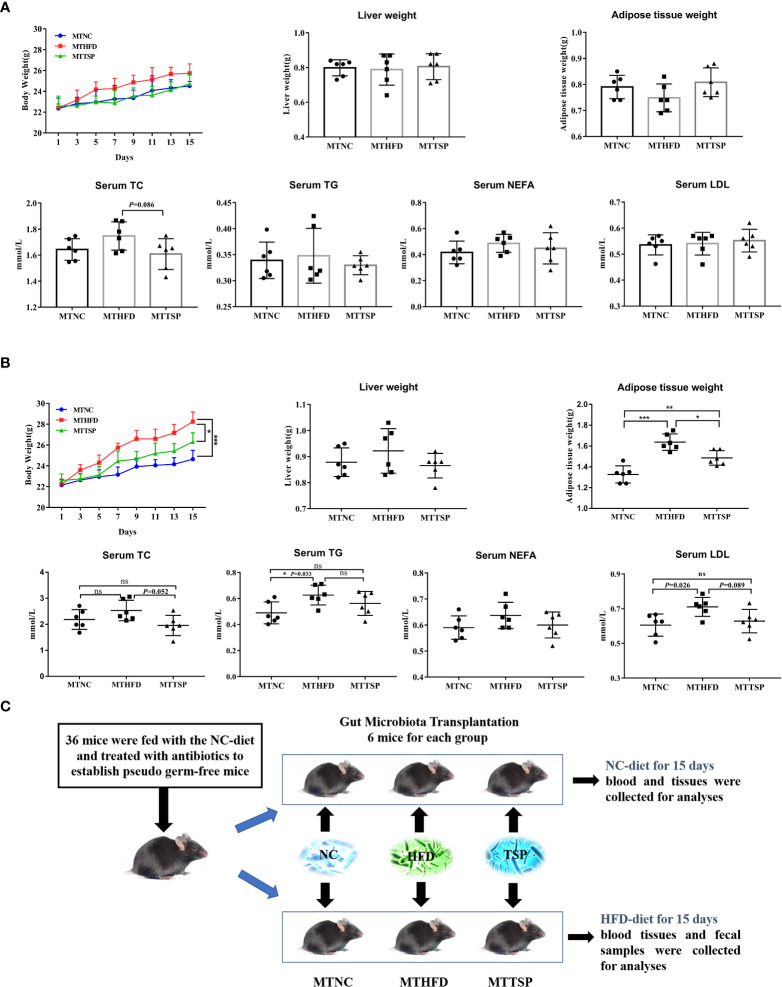
Microbiota Transplantation from TSP- supplementation Mice Exerts an Anti-Obese Effect in HFD-Fed Mice. **(A)** Microbiota-transplanted mice fed with NC-diet; **(B)** microbiota-transplanted mice fed with HFD-diet; **(C)** The experiment design of microbiota transplantation. Data are presented as mean ± SEM, differences were denoted as follows: *p < 0.05, **p < 0.01, ***p < 0.001; ns, no significant *p* > 0.05.

The fecal bacteria from NC-, HFD-, or TSP-fed mice (ten weeks) were transplanted to pseudo germ-free mice to explore whether TSP supplementation could attenuate hyperlipidemia in HFD-diet mice by altering gut microbiota (**Figure 9**). As revealed in **Figure 9A**, after microbiota transplantation, the weight of body, liver and adipose tissue and the indexes of lipid metabolism in serum (TC, TG, NEFA, and LDL) in mice fed with NC-diet have no significant different (*p* > 0.05).

It had different results in microbiota-transplanted mice fed with HFD (**Figure 9B**). Compared with MTHFD group, the weight of body and adipose tissue in MTNC and MTTSP mice was significantly decreased (*p* < 0.05). Furthermore, MTNC group mice significantly reduced the serum content of TG and LDL compared to MTHFD group. However, there was no significant different in the liver weight and serum levels of TC and NEFA among the three groups (*p* > 0.05). The detailed experimental scheme is shown in **Figure 9C**.

**Figure 10:**
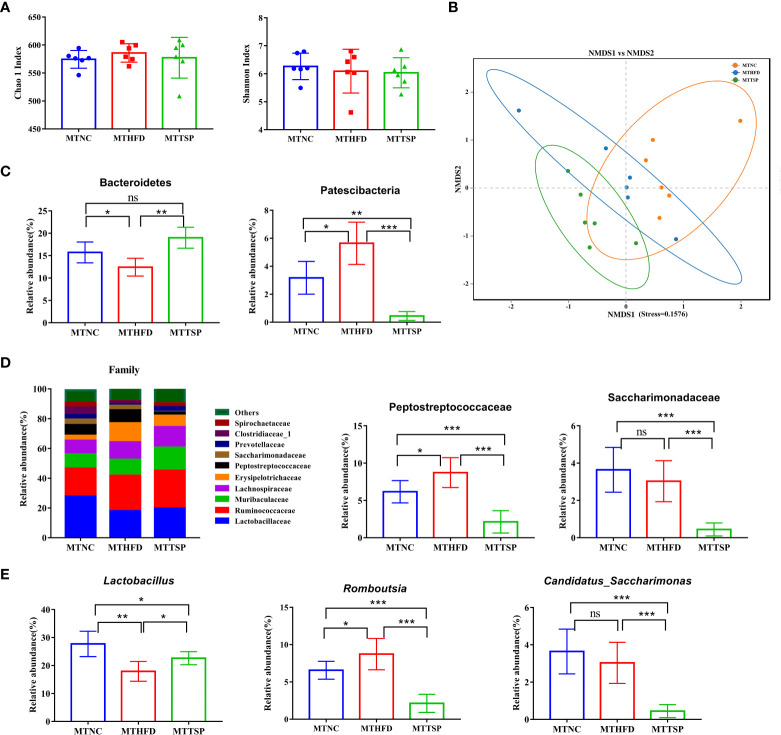
Gut microbiota in response to microbiota transplantation from NC (MTNC), HFD (MTHFD), and TSP (MTTSP) groups (n = 6), **(A)** Indexes of Chao 1 and Shannon in α-diversity analysis, **(B)** NMDS plot analysis from each sample, **(C)** significantly changes of the composition of the gut microbiota at phylum taxa level (*p* < 0.05), **(D)** microbiota compositions at the family level, and **(E)** significantly changes of the composition of the gut microbiota at the genus level (*p* < 0.05). Data are expressed as mean ± SD, *p **p ***p/span>, ns, no significant, *p* > 0.05.

Then, the gut microbiota phylotypes of microbiota-transplanted mice fed with HFD-diet were further measured by sequencing the bacterial 16S rRNA. As revealed in **Figure 10A**, no significant different was observed in alpha diversity among all groups. Furthermore, we analyzed β diversity which indicate the gut microbiota structural changes by using the NMDS on the Bray-Curtis algorithm (**Figure 10B**) showed significant different among the three groups (*p*< 0.01, R^2^ = 0.26, Stress=0.1576). At the phylum level, the alterations in the relative abundances of Bacteroidetes and Patescibacteria in MTHFD mice showed the same trends as it of HFD-fed mice (**Figure 10C**). Meanwhile, the relative abundance of Peptostreptococcaceae at the family level in MTNC and MTTSP mice tended to decrease relative to the MTHFD mice (*p* < 0.05), whereas the relative abundance of Saccharimonadaceae in MTTSP group significantly decreased compared with MTNC and MTHFD group (*p* < 0.05, **Figure 10D**). Additionally, the relative abundance of *Lactabacillus* and *Romboutsia* at the genus level was significantly changed among the three groups (*p*< 0.05, **Figure 10E**), and they also showed the same trends as it of HFD-fed mice. The relative abundance of *Candidatus_Saccharimonas* in MTTSP group was significantly reduced compared with MTNC and MTHFD group (*p* < 0.01). Overall, these data indicate that mechanism of TSP to inhibit the occurrence of lipid metabolism disorder and obesity may be realized by regulating intestinal microbiome.

## Discussion

*Tolypocldium sinenis* is an entomogenous fungus isolated from the mycelial tissue of the sclerotia and cotyledon of Chinese caterpillar fungus. Some gene sequencing results of TSP are compared with the gene sequencing results of *Cordyceps sinensis* (accession number AF291749) in the gene database, and the similarity is 99% (28). Relevant studies showed that TSP and *C. sinensis* have basically the same pharmacological effects, indicating that TSP has a potential pharmacological value. At present, some reports on the bacteriostatic, anti-inflammatory, antioxidative stress and anti-tumor effects of TSP are available, but the effect of TSP on preventing obesity and on the change of the intestinal microbiota has not been studied. The regulation of the composition of the gut microbiota is a promising approach to prevent the development of obesity and related metabolic disorders. This study was the first showing that the dietary supplementation TSP prevented HFD-induced obesity and hyperlipemia. The potential mechanism could reduce systemic inflammation and by regulating the composition and potential function of the gut microbiota.

As a global epidemic, obesity increases the risk of a variety of chronic diseases, reduces life expectancy and brings a serious personal and socio-economic burden (29–31). Different theoretical explanations in obesity research are available. One of the important reasons is the change of people’s diet, since the high-fat diet is an important factor in obesity. The excessive weight gain leads to the abnormal increase in blood lipid and blood glucose levels (32). Our results also revealed that HFD feeding promoted an evident increase in body weight, liver weight, serum lipid levels, and fat vacuoles in hepatocytes in mice when compared with these parameters in the NC group, which was in agreement with previously published reports (33, 34). TSP supplementation reduced the accumulation of abdominal adipose tissue induced by HFD and effectively prevented the increase in body weight and liver weight in mice. Moreover, TSP decreased the levels of TC, TG and NFFA in serum and liver, and prevented the increase of serum LDL-C and TBA in liver, indicating its potential preventive effect on the development of fatty liver disease induced by HFD. However, our results revealed that food consumption and energy intake of mice supplemented with TSP were similar to those in the HFD group, indicating that the role of TSP in preventing obesity was not related to the reduction of appetite.

The genes related to liver lipid/cholesterol synthesis and metabolism were measured by qRT-PCR to further clarify how TSP supplementation prevented HFD feeding-induced liver lipid metabolism disorder. The results revealed that TSP supplement down-regulated the expression of ACC, HMGCR, LXRα and SREBP-1c, which involved in hepatic lipid/cholesterol synthesis and metabolism (35), and up-regulated the expression of AMPK and PPARα, that involved in fatty acid oxidation (36, 37), compared with their expression in the HFD group. HMGCR catalyzes 3-hydroxyl in the process of cholesterol synthesis 3-methylglutaryl CoA is the rate limiting enzyme for the conversion of 3-methylglutaryl CoA to mevalonate (37). Therefore, the inhibition of the activity of HMGCR in the liver reduces the synthesis of cholesterol in the body, thus regulating the disorder of lipid metabolism. SREBPs promotes the regulation of cholesterol and adipose formation by a strict transcription and post-translational regulation. SREBP1c is an important subtype of SREBPs that positively regulates cholesterol and fatty acid syntheses and uptake in the hepatocytes (38). PPARα is one of the PPAR family proteins, and play an important role in lipid metabolism, glucose homeostasis and anti-inflammatory effects, and upregulation the mRNA level of PPARα expression could promote fatty acid catabolism and reduce fat mass (7, 39, 40). PPARα enhances the antioxidant function of hepatocytes by regulating the levels of SOD, ALT and AST (41). In addition, PPARα reduces serum cholesterol and LDL levels by regulating cholesterol 7A-hydroxylase, sterol 12a-hydroxylase, increases the level of high-density lipoprotein (HDL), hydrolyzes very low-density lipoprotein (VLDL), and delays the progression of coronary atherosclerosis (42). Thus, TSP supplementation might partially contribute to the regulation of genes of the lipid metabolism involved genes in liver, preventing adipose tissue deposition and improved hyperlipidemia in HFD mice.

Studies showed that chronic obesity is closely related to low-grade inflammation. Obesity-induced inflammation is called metabolic inflammation, which is different from the classical inflammation because the metabolic inflammation belongs to the chronic and low-grade inflammation (43). Mice fed with long-term HFD have hyperglycemia, hyperlipidemia, as well as increased systemic chronic inflammation and proinflammatory factors (44–46). Our results showed that HFD feeding promoted the occurrence of inflammation in the serum and liver tissue, and the daily supplement of TSP effectively inhibited the inflammatory factors (TNF-α, IL-6, and IL-1β). Some studies reported that oxidative stress is a part of the inflammatory response, which activates the cascade of inflammatory signals to promote the occurrence of inflammation. Furthermore, the oxygen free radicals produced by inflammatory activated immune cells further aggravate the oxidative stress response (47). Obese people are often accompanied by impaired mitochondrial function, such as decreased mitochondrial density and ATP synthesis (48). It is reported that mitochondrial dysfunction precedes hepatic steatosis and insulin resistance in obese rodent model (49),. The increased level of circulating fatty acids in obese individuals leads to the excessive accumulation of lipids in cells, the damage of the mitochondrial function and the increase in the content of ROS. Excessive ROS not only damages the ability of mitochondrial ATP synthesis and oxidative phosphorylation, but also interferes with the replication of mitochondrial DNA and RNA, affecting the structure and function of mitochondria, and leading to mitochondrial dysfunction and further increase in the production of ROS (50). Relevant studies confirmed that the mycelial extract and mycelial polysaccharide of TSP have sedative, anti-inflammatory and antioxidant effects (16, 18, 51), which might be due to the effect of cyclosporin A rich in TSP. Cyclosporin A is widely used as an immunosuppressant to avoid rejection of organs after transplantation, and has a certain antifungal and anti-inflammatory effects (52, 53). Our study found that TSP supplementation effectively inhibited the increase of MDA level in the serum and liver caused by HFD, as well as it significantly increased the SOD and GSH-Px activity.

Different dietary structure can modify and change the composition and structure of the intestinal flora, so as to change the physiological metabolism of the host. As a bioreactor of human food, the intestinal microorganisms are related to many physiological effects and diseases of the host, especially obesity (54, 55). More and more studies showed that intestinal microorganisms can affect host metabolism through immune, endocrine and intestinal brain axis (56–58), so as to regulate human energy absorption, lipid metabolism and inflammatory response. For example, the gut microbiota metabolizes complex carbohydrates and plant polysaccharides to produce short chain fatty acids, which are an important substrate providing energy for the human body and microorganisms (59). It mainly includes acetic acid, butyric acid, and propionic acid. In addition to being a direct energy supplier, short chain fatty acids also play a role in metabolic regulation after entering the tissues and organs by blood circulation, working as signal molecules by stimulating the release of saturated hormone peptides (60) and glucagon peptides (61, 62). The intestine by activating nutrient receptors to reduce physical inflammation and brain signal transmission. Previous studies showed that dietary short chain fatty acid supplementation is effective in preventing obesity and dyslipidemia in HFD-fed mice (63, 64). TSP supplementation partially restored the HFD-induced decrease in SCFAs, especially the content of butyrate was significantly higher than that of the blank control, while acetate and propionate went back to normal levels. Butyric acid strengthens the intestinal barrier by affecting the length of small intestinal villi and mucosal thickening to control the occurrence of metabolic diseases (65).

Since the gut microbiota serves as a pivotal mediator in the regulation of host energy absorption, appetite and consumption (66), our study also found that the alpha diversity of gut microbiota was significantly lower in the HFD group than that in the NC group. Generally, dietary supplementation or weight loss contribute to the recovery of gut microbial diversity (67). Our results showed that although TSP supplementation inhibited weight gain, it did not restore gut microbial diversity, which might be related to its pharmacological properties. The efficacy of TSP is associated to immune regulation, anti-tumor and antioxidant effect, as well as bacteriostasis (68). Therefore, TSP supplementation might inhibit some non-probiotics and reduce the gut microbial diversity. Our study demonstrated that TSP supplementation could alter the gut microbiota structure and composition revealed by NMDS and hierarchical cluster analysis. TSP supplementation did not affect the relative abundance of Firmicutes, but it significantly enhanced the relative abundance of Bacteroidetes, thus significantly decreasing the F/B ratio. The higher F/B ratio in the intestines of obese individuals could promote obesity of the host by absorbing energy from food (69). Wu et al (70). suggested that reducing the HFD-induced increase of the F/B ratio together with the reduction of the inflammatory markers IL-2, IL-6 and TNF-a in mice serum could be obtained by promoting the growth of Bacteroidetes. TSP also significantly inhibited the proliferation of Proteobacteria and Patescibacteria. Studies suggested that the proportion of Bacteroidetes in the gut microbiota of NAFLD patients was lower than non-obese people, accompanied by an increase in Actinomycetes, Firmicutes and Proteobacteria and the abnormal increase of Proteobacteria in gut microbiota reflects the imbalance of microecology or the instability of gut microbiota structure (71, 72). The specific families reported as increased by HFD include Lactobacillaceae, Ruminococcaceae, Lachnospiraceae and Clostridiaceae (70). Our results showed that daily supplementation of TSP effectively and significantly reduced the relative abundance Ruminococcaceae and Clostridiaceae compared with the HFD mice. On the contrary, TSP mice showed a higher and significant relative abundance of Lactobacillaceae than HFD mice. *Lactobacillus* is traditional probiotics that play an important role in the balance of the human intestinal microecology. Some studies reported that the increase in *Lactobacillus* effectively reduces serum cholesterol level and body fat (73), and it also prevents chronic inflammation and the worsening of insulin resistance and are recognized as beneficial bacteria (74, 75). The correlation analysis showed that *Lactobacillus* was significantly negatively correlated with inflammatory factors and lipid metabolism in our study. The comprehensive analysis revealed that the HFD group showed a higher abundant of *Romboutsia* and *Candidatus_Saccharimonas* which positively correlated with obesity and obesity-related physiological markers. However, TSP group mice had significantly lower abundance of bacteria linked to obesity than HFD-fed mice. *Romboutsia* are linked with obese like features and they are highly abundant in obese mice (76–78). The spearman correlation analysis also found that *Romboutsia* were positively correlated with serum and liver inflammatory factors in our study. In some ways, TSP supplementation also effectively increased the relative abundance of the two species *Prevotella_9* and *Allobaculum*. The abundance of genera *Prevotella* is associated with carbohydrate intake (79). A recent study further revealed that individuals with a high abundance of *Prevotella* were more likely to lose weight than those with *Bacteroides*, when these individuals go on a diet (80). To summarize our discovery, our work demonstrated that the beneficial bacteria that protects the body increased after TSP supplementation and it protects the balance of the gut microbiota from being destroyed by high-fat diet, inhibiting the growth of bacteria that promote obesity, and improving the relative abundance of beneficial bacteria.

The change of microbial composition is always accompanied with significant functional alteration and changes in the gut microbiota that inevitably lead to changes in the host metabolism (81, 82). Therefore, in this study, the functional abilities of the microbial communities were analyzed by PICRUSt. The reduced lipid accumulation in the liver of TSP mice might be caused by the functions related to carbohydrate and lipid metabolism in microbiota. TSP effectively enhanced the carbohydrate and lipid metabolism ability, suggesting that the microbiome in TSP mice might consume more dietary lipid and more carbohydrates. These findings are also consistent with the more profound downregulation of lipogenic gene expressions by TSP treatment. Combining with the existing results, our hypothesis was that the effects of TSP on attenuating HFD-induced lipid metabolism disorders were mediated, at least in part, by the modulation of the gut microbiota. We confirmed this hypothesis with the fecal bacteria transplant experiment, that gut microbiota might be required for TSP to carry out its anti-obese and prevent hyperlipidemia effects on HFD-fed mice.

## Conclusion

In summary, TSP supplementation confers protective effects against HFD-induced obese and hyperlipidemia by altering the gut microbiota, alleviate the inflammatory response and regulating gene expression of liver lipid metabolism. It raises the possibility that TSP poses great therapeutic potential in treating obesity and its complications. Therefore, the development of TSP act as daily health food has a good prospect.

## Data availability statement

The datasets presented in this study can be found in National Microbiology Data Center (https://nmdc.cn/), with the Accession number: NMDCX0000149.

## Ethics statement

The animal study was reviewed and approved by the Animal Ethics Committee of the Qiqihar Medical University

## Author contributions

CZ, XW, HY and XC designed the experiments. XW, and MB performed the animal experiments. XW and LL conducted gut microbiota analysis. XW, LL, JZ, YG, and XS measured biochemical data and conducted immunohistochemical analysis. XW and LL wrote the draft of the manuscript. All authors contributed to the article and approved the submitted version.

## Funding

This work was funded by the Qiqihar Medical University, grant number QMSI2017B-06, QMSI2017B-13 and QMSI2019L-27, by Natural Science Foundation of Heilongjiang Province, grant number LH2020H129, by Health commission of Heilongjiang Province, grant number 20210101060181 and by Graduate Innovation Fund of Qiqihar Medical University, grant number QYYCX2022-05.

## Conflict of interest

The authors declare that the research was conducted in the absence of any commercial or financial relationships that could be construed as a potential conflict of interest.

## Publisher’s note

All claims expressed in this article are solely those of the authors and do not necessarily represent those of their affiliated organizations, or those of the publisher, the editors and the reviewers. Any product that may be evaluated in this article, or claim that may be made by its manufacturer, is not guaranteed or endorsed by the publisher.
