# Supplementary material for: Dietary supplementation with Tolypocladium sinense mycelium prevents dyslipidemia inflammation in high fat diet mice by modulation of gut microbiota in mice
Source: Front Immunol. 2022 Nov 7;13:977528. doi: 10.3389/fimmu.2022.977528 (PMC9677100; doi:10.3389/fimmu.2022.977528)
Supplement: Supplementary file 1 [file DataSheet_1.docx]

**Supplementary information**

**Dietary Supplementation with *Tolypocladium sinense* Powder Prevents Dyslipidemia and Inflammation in High Fat Diet Mice by Modulation of Gut Microbiota in Mice**

Xiaolong Wang^1†^, Lin Li^1†^, Mingjian Bai^1^, Jiaxin Zhao^2^, Xiaojie Sun^1^, Yu Gao^1^, Chunjing Zhang^1*^, Xia Chen^2*^

1 Department of Medical Technology, Qiqihar Medical University, Qiqihar 161006, Heilongjiang, China

2 National & Local United Engineering Laboratory for Chinese Herbal Medicine Breeding and Cultivation, Schoolof Life Sciences, Jilin University, Changchun 130012, China.

***** Correspondence: cjzhang2005@163.com (C.-j. Z.); chenxiajlu@163.com (X. C.);

Tel.: +86-0452-266-3381(C.-j. Z.); +86-0431-85155284 (X. C.)

† These authors contributed equally to this work.

**Supplemental methods**

**1.** **UHPLC-QTOF-MS non target metabolomics detection for TSP**

*Tolypocladium sinense* (TSP) powder Metabolite extraction: weigh *Tolypocladium sinense* powder 50 mg of sample and add 1000 mg μL extraction solution containing internal standard (1000:2) (methanol acetonitrile water volume ratio = 2:2:1, internal standard concentration 2 mg/L), vortex mixing for 30 seconds; Add ceramic beads, treat with 45Hz grinder for 10min, and ultrasonic for 10min (ice water bath); Stand at minus 20 ℃ for one hour; Centrifuge the sample at 4 ℃ 12000rpm for 15min; Carefully take out 500 μL supernatant in EP pipe; Dry the extract in a vacuum concentrator; Add 160 to the dried metabolite μL extraction solution (volume ratio of acetonitrile to water: 1:1) was re dissolved; Vortex for 30 seconds, ice water bath ultrasonic for 10 minutes; Centrifuge the sample at 4 ℃ 12000rpm for 15min; Carefully take out 120 μL supernatant in 2mL injection bottle, take 10 samples for each sample 10 μL mixed into QC samples for machine testing.

The LC-MS system for metabonomic analysis is composed of wattage acquisition I-class plus ultra-high performance liquid chromatography in series with wattage Xevo g2-xs QTOF high resolution mass spectrometer. The chromatographic column used is acquisition UPLC HSS T3 chromatographic column (1.8um, 2.1 * 100mm) purchased from wattage.

Positive ion mode: mobile phase A: 0.1% formic acid aqueous solution; Mobile phase B: 0.1% formic acid acetonitrile; Negative ion mode: mobile phase A: 0.1% formic acid aqueous solution; Mobile phase B: 0.1% formic acid acetonitrile, Injection volume 1ul.

Mass spectrum conditions: Water Xevo G2-XS QTof high resolution mass spectrometer can collect primary and secondary mass spectrometry data in MSE mode under the control of acquisition software (MassLynx V4.2, Waters). In each data acquisition cycle, dual channel data acquisition can be carried out for low collision energy and high collision energy at the same time. The low collision energy is 2V, the high collision energy range is 10 ~ 40V, and the scanning frequency is 0.2s. ESI ion source parameters are as follows: capillary voltage: 2000V (positive ion mode) or - 1500V (negative ion mode); Taper hole voltage: 30V; Ion source temperature: 150 ℃; Desolvent gas temperature 500 ℃; Blowback flow rate: 50L/h; Flow rate of desolvent gas: 800L/h, chromatographic test results see Figure S1.

Data processing: Use MassLynx V4.2 collected raw data are processed by the progenesis QI software for peak extraction, peak alignment and other data processing operations, and identified based on the online METLIN database of the Progenesis QI software and the self built database of BioMarker Technology. At the same time, the theoretical fragments are identified, and the mass number deviation is within 100ppm.

**2.** **Preliminary animal experiment**

A preliminary animal experiment was conducted to obtain the desired TSP. Forty male C57BL/6 weighing 20 ± 1g provided by the experimental animal center of Qiqihar Medical College (SYXK (HEI) 2016-001) were assigned to five experimental groups for 8 weeks (n = 8 for each group): NC group, mice were fed normal diet (total calories: 4.3 kcal/g, 10 kcal% fat); HFD group, mice were fed high-fat diet (total calories: 6.1 kcal/g, 60 kcal% fat); mice fed a high fat diet with daily oral gavage of TSP (100 mg/kg/day HFD+LTSP), mice fed a high fat diet with daily oral gavage of TSP (150 mg/kg/day HFD+MTSP), mice were fed high-fat diet; mice fed a high fat diet with daily oral gavage of TSP (300 mg/kg/day HFD+HTSP). Body weight was monitored once a week. At the end of the 8th week, mice were fasted overnight and sacriﬁced, the whole blood of rats was collected, the serum was taken after centrifugation and perirenal fat pads, livers, and gastrointestinal tract were collected and weighed. The fasting blood glucose, serum total cholesterol (TC), triglyceride (TG), high density lipoprotein cholesterol (HDL-C), low density lipoprotein cholesterol (LDL-C) and non-esterified fatty acid (NEFA) were measured in strict accordance with the instructions of the kit.

**Supplemental tables**

Table S1 Sequences of primers used for qPCR in this study.

| Name | Sequence（5'-3') |
| --- | --- |
| ITS1 | TCCGTAGGTGAACCTGCGG |
| ITS4 | TCCTCCGCTTATTGATATGC |
| β-actin | F: GTGACGTTGACATCCGTAAAGA |
|  | R: GTAACAGTCCGCCTAGAAGCAC |
| GAPDH | F: CCTCGTCCCGTAGACAAAATG |
|  | R: TGAGGTCAATGAAGGGGTCGT |
| AMPK | F: ATCCGAGGGGGTGTGTTCTA |
|  | R: TAGTTGCTCGCTTCAAGGGG |
| ACC | F: TTCAGTTCATGCTGCCCACA |
|  | R: AGGTTGGAGGCAAAGGACAT |
| FAS | F: TGAATCAGCCCCACGCAGT |
|  | R: CCGAGTCAGTCTTGGAGGACAT |
| PPARα | F: GAGGCAGATGACCTGGAAAGT |
|  | R: TGCGTGAACTCCGTAGTGGTA |
| PPARγ | F: CCATTCTGGCCCACCAAC |
|  | R: AATGCGAGTGGTCTTCCATCA |
| SREBP-1c | F: CACTTCTGGAGACATCGCAAAC |
|  | R: GTCCTCCTGTGTACTTGCCCA |
| HMGCR | F: AGTGGTGCGTCTTCCTCG |
|  | R: CGAATCTGCTGGTGCTAT |
| CD36 | F: GACAATCAAAAGGGAAGTTG |
|  | R: CCTCTCTGTTTAACCTTGAT |
| LDLR | F: TGGCTATGAGTGCCTATGTCC |
|  | R: GGTGAAGAGCAGAAACCCTATG |
| CYP7A1 | F: CACCATTCCTGCAACCTTTT |
|  | R: GTACCGGCAGGTCATTCAGT |
| LXRα | F: TCAGAAGAACAGATCCGCTTG |
|  | R: CGCCTGTTACACTGTTGCT |
| LXRβ | F: GATCCTCCTCCAGGCTCTGAA |
|  | R: TGCGCTCAGGCTCATCCT |

Table S2 Effects of different dose of TSP in preliminary animal experiment

| Parameter | ND | HFD | HFD+LTSP | HFD+MTSP | HFD+HTSP |  |
| --- | --- | --- | --- | --- | --- | --- |
|  |  |  |  |  |  |  |
| Body weight gain (g) | 6.85±0.74 | 16.51±3.44^**^ | 14.85±4.21 | 13.49±1.33* | 11.43±2.25^##^ |  |
| Adipose tissue weight | 0.99±0.11 | 3.01±0.23^**^ | 2.98±0.08 | 2.47±0.13^##^ | 2.30±0.11^##^ |  |
| Liver weight (g) | 0.88±0.16 | 1.32±0.15^**^ | 1.30±0.09 | 1.09±0.11^#^ | 1.01±0.18^#^ |  |
| Fasting glucose (mmol/L) | 6.12±1.50 | 11.89±0.47^**^ | 10.11±1.28^*^ | 8.78±2.36^#^ | 9.05±2.16^#^ |  |
| TC (mmol/L) | 3.04±0.67 | 6.12±0.85^**^ | 6.01±0.69 | 5.22±1.01^#^ | 4.68±0.63^##^ |  |
| TG (mmol/L) | 0.70±0.09 | 0.94±0.11^*^ | 0.95±0.07 | 0.88±0.12 | 0.77±0.09^#^ |  |
| HDL-C (mmol/L) | 1.57±0.34 | 2.25±0.37^**^ | 2.17±0.23 | 2.42±0.41 | 2.41±0.34 |  |
| LDL-C (mmol/L) | 1.06±0.40 | 2.15±0.36^**^ | 2.03±0.44 | 1.98±0.37 | 1.68±0.23^##^ |  |

Note: Values are represented as mean *±* standard deviations, compared with NC group, * *p* < 0.05, ***p* < 0.01; Compared with HFD, ^#^ *p* < 0.05, ^##^*p* < 0.01

Table S3 Changes of the composition of the gut microbiota at family taxa level.

|  | NC | HFD | HFD+TOS |
| --- | --- | --- | --- |
| Lactobacillaceae | 31.53±13.63a | 18.36±5.48b | 35.7±16.44 a |
| Ruminococcaceae | 16.62±5.39 ab | 21.71±7.18 b | 12.79±4.10 a |
| Muribaculaceae | 17.22±5.02a | 10.70±2.88b | 13.04±2.69a |
| Lachnospiraceae | 8.52±3.80a | 13.48±6.54a | 13.85±5.83a |
| Erysipelotrichaceae | 4.23±4.92a | 6.49±5.60a | 9.05±3.61b |
| Peptostreptococcaceae | 3.58±2.53a | 9.24±3.30c | 0.21±0.23b |
| Prevotellaceae | 6.04±3.16a | 1.18±0.43b | 4.80±a |
| Spirochaetaceae | 0.84±1.38a | 2.10±2.31a | 4.00±5.28a |
| Clostridiaceae_1 | 0.88±0.94a | 4.52±5.26b | 0.0059±0.006a |
| Saccharimonadaceae | 0.43±0.24a | 3.55±1.15b | 0.23±0.24a |

Data are expressed as mean ± SD. Values with different letters are significantly different (*p* < 0.05).

**Supplemental figures**


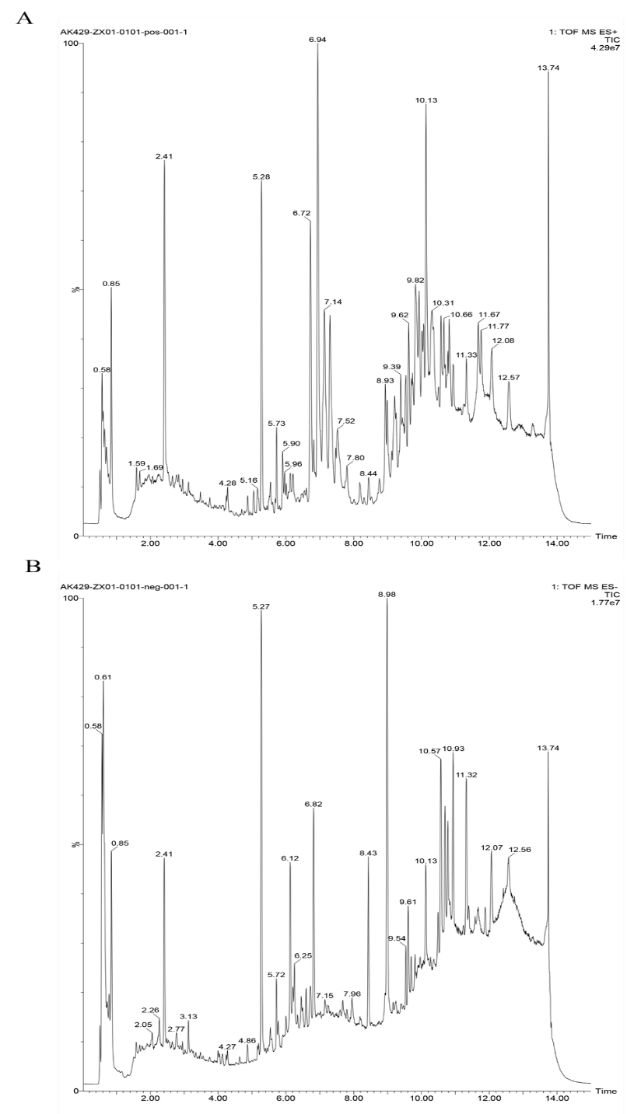


**Figure S1. The result of UHPLC-QTOF-MS non target metabolomics detection of TSP**

**
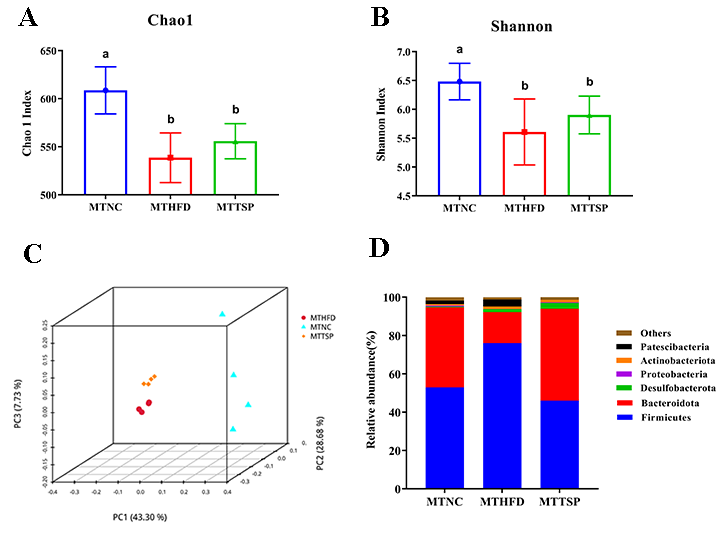
**

**Figure S2.** **Analysis of intestinal microbiota after fecal bacteria transplantation and colonization. (A) Alpha diversity analysis of Chao1 and (B) Shannon index (C) PCoA result based on Bray Curtis algorithm. (D) the composition of the gut microbiota at phylum taxa level.**


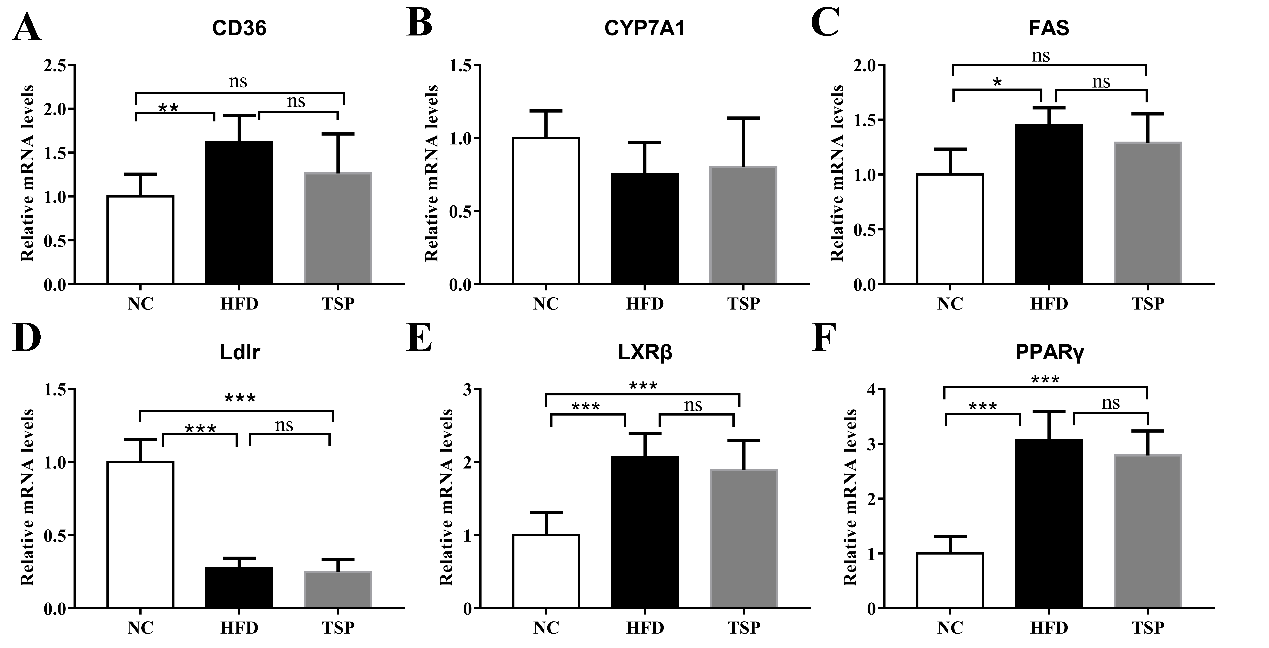


**Figure S**3 Effect of TSP on mRNA expression levels of hepatic metabolic regulators. (A) CD36 (B) CYP7A1, (C) FAS, (D)Ldlγ, (E) LXRβ, (F) PPARγ. Data are expressed as means ± SD (n = 8), * *p* < 0.05, ** *p* < 0.01, and *** *p* < 0.001.


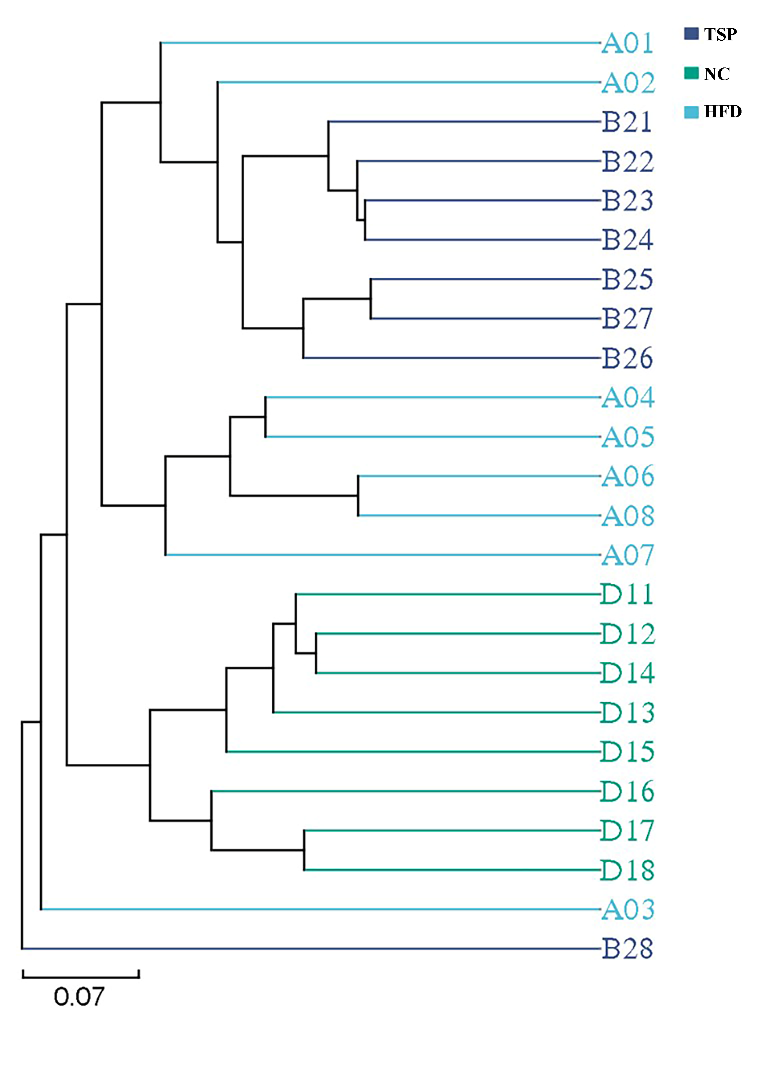


**Figure S**4 UPGMA analysis based on different groups.
